# The analysis on groundwater storage variations from GRACE/GRACE-FO in recent 20 years driven by influencing factors and prediction in Shandong Province, China

**DOI:** 10.1038/s41598-024-55588-3

**Published:** 2024-03-09

**Authors:** Wanqiu Li, Lifeng Bao, Guobiao Yao, Fengwei Wang, Qiuying Guo, Jie Zhu, Jinjie Zhu, Zhiwei Wang, Jingxue Bi, Chengcheng Zhu, Yulong Zhong, Shanbo Lu

**Affiliations:** 1 School of Surveying and Geo-Informatics, Shandong Jianzhu University, Jinan, 250101 China; 2 State Key Laboratory of Geodesy and Earth’s Dynamics, Innovation Academy for Precision Measurement Science and Technology, CAS, Wuhan, 430077 China; 3 College of Surveying and Geo-Informatics, Tongji University, Shanghai, 200092 China; 4 China Earthquake Networks Center, Beijing, 100045 China; 5 801 Institute of Hydrogeology and Engineering Geology, Shandong Provincial Bureau of Geology & Mineral Resources, Jinan, 250014 China; 6 School of Geography and Information Engineering, China University of Geosciences (Wuhan), Wuhan, 430074 China

**Keywords:** GRACE, GWS, ICA, Influencing factors, SVM, Prediction, Shandong Province, Hydrology, Solid Earth sciences

## Abstract

Monitoring and predicting the regional groundwater storage (GWS) fluctuation is an essential support for effectively managing water resources. Therefore, taking Shandong Province as an example, the data from Gravity Recovery and Climate Experiment (GRACE) and GRACE Follow-On (GRACE-FO) is used to invert GWS fluctuation from January 2003 to December 2022 together with Watergap Global Hydrological Model (WGHM), in-situ groundwater volume and level data. The spatio-temporal characteristics are decomposed using Independent Components Analysis (ICA), and the impact factors, such as precipitation and human activities, which are also analyzed. To predict the short-time changes of GWS, the Support Vector Machines (SVM) is adopted together with three commonly used methods Long Short-Term Memory (LSTM), Singular Spectrum Analysis (SSA), Auto-Regressive Moving Average Model (ARMA), as the comparison. The results show that: (1) The loss intensity of western GWS is significantly greater than those in coastal areas. From 2003 to 2006, GWS increased sharply; during 2007 to 2014, there exists a loss rate − 5.80 ± 2.28 mm/a of GWS; the linear trend of GWS change is − 5.39 ± 3.65 mm/a from 2015 to 2022, may be mainly due to the effect of South-to-North Water Diversion Project. The correlation coefficient between GRACE and WGHM is 0.67, which is consistent with in-situ groundwater volume and level. (2) The GWS has higher positive correlation with monthly Global Precipitation Climatology Project (GPCP) considering time delay after moving average, which has the similar energy spectrum depending on Continuous Wavelet Transform (CWT) method. In addition, the influencing facotrs on annual GWS fluctuation are analyzed, the correlation coefficient between GWS and in-situ data including the consumption of groundwater mining, farmland irrigation is 0.80, 0.71, respectively. (3) For the GWS prediction, SVM method is adopted to analyze, three training samples with 180, 204 and 228 months are established with the goodness-of-fit all higher than 0.97. The correlation coefficients are 0.56, 0.75, 0.68; *RMSE* is 5.26, 4.42, 5.65 mm; *NSE* is 0.28, 0.43, 0.36, respectively. The performance of SVM model is better than the other methods for the short-term prediction.

## Introduction

Groundwater Storage (GWS) resources are an essential part of global water cycle system and one of the most critical issues concerning to the country's economic and social development^1–3^. The traditional methods of monitoring GWS change include pressure gauge and ground network measurement. The groundwater model can also estimate regional GWS change, but it has excellent limitations for model description and data acquisition. The launch of GRACE could provide an essential opportunity for monitoring global and regional GWS change^4,5^.

At present, it has been successfully applied in many regions of China, such as North China Plain, Huang-Huai-Hai Plain, Songhua River Basin Northwestern China, Southwest China, etc. These studies mainly focus on the overall changes of GWS and the consistency analysis on trends with precipitation. However, the detail correlation characteristic on the time series of rainfall and GWS change from GRACE needs to further study. Furthermore, the impact of human activities on GWS change is not considered in most studies, and the measured shallow groundwater level data is used to verify for GRACE data. Still, the in-situ data reflecting the regional GWS change is lacking. Shandong Province is considered as a significant agricultural province in China, which locates in the lower reaches of the Yellow River, and plays a vital role in the eastern route for South-to-North Water Diversion Project. Therefore, the GWS change driven by climate change and human activities in Shandong Province is complex. Monitoring the GWS change in Shandong province over the recent 20 years, and investigating the causes are of great significance to the management and sustainable development of regional water resources.

Additionally, accurately predicting the GWS change can provide an important reference for water resources planning and management. However, the instability and mutability for the time series of GWS changes, as well as the small-sample characteristics of GRACE data, which can bring some difficulties to accurately prediction^6^. The prediction of time series is one of the leading research topics in artificial intelligence^7–10^. How to establish a training model depending on small samples for GRACE data to achieve higher-precision prediction is an important content in the entire water resources field. There are standard methods on predicting for the time series, such as least squares fitting, SSA, ARMA, neural network^11–14^. Statistical learning is a machine learning theory for small samples, aiming to control the generalization ability by managing the complexity of learning machine^15–18^. The SVM method is developed under the theory of Vapnik–Chervonenkis (VC) Dimension and Structural Risk Minimization. This method can solve practical problems such as small samples, over-learning, nonlinear, high-dimensional, etc.

GRACE data combined with the Global Land Data Assimilation System (GLDAS) hydrological model are used to invert GWS changes. Most of the domestic research focuses on the analysis of spatio-temporal evolution characteristics of typical regions^19–23^, such as North China, Tianshan Mountains, Liaohe River Basin, etc., while the long-term series of GWS changes in Shandong Province and its prediction research is relatively scarce. Therefore, the changes of GWS in Shandong Province in recent 20 years is focused on this paper. Piecewise linear fitting and continuous wavelet transform (CWT) methods are adopted to analyze the detailed characteristics. Multi-type data are introduced, such as Watergap Global Hydrological Model (WGHM) model, GPCP precipitation model, measured groundwater exploitation and groundwater level data. The inversion results are comprehensively verified, and the influence of precipitation and human factors is deeply analyzed. The SVM machine learning algorithm is used to model the long time series of GWS and predict the changes of GWS.

It should be noted that meteorological data such as precipitation, temperature, and evapotranspiration are not introduced as constraints in the process of sample training, but only the time series of GWS derived from GRACE data with about 330 km spatial resolution are adopted, and artificial intelligence algorithms such as SVM method are used for prediction and analysis, the accuracy of which is evaluated, comparing with that from LSTM, SSA, ARMA methods.

## Data

### The study area

**Figure 1:**
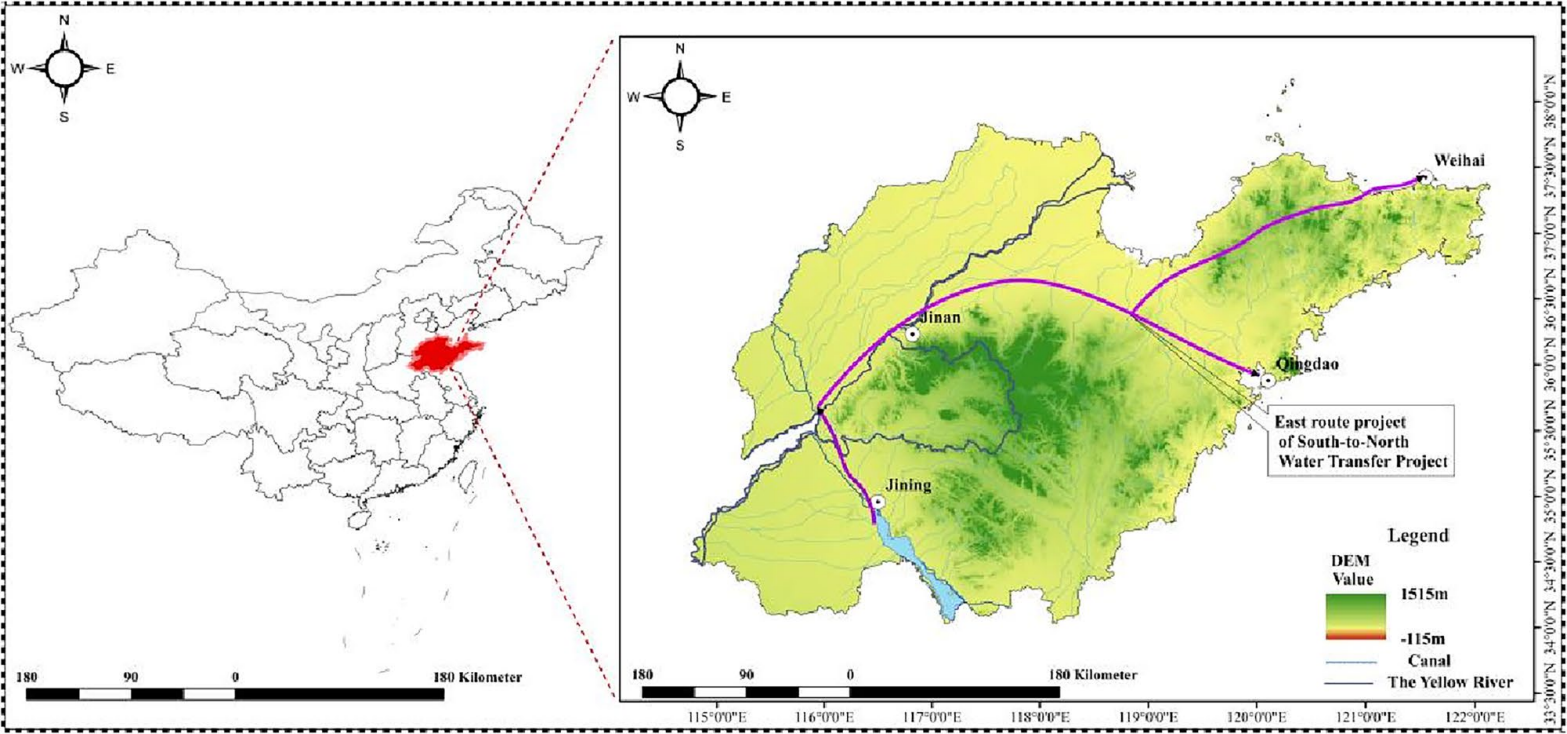
The regional position picture of Shandong Province (It was generated by ArcMap 10.8.2 software (https://www.esri.com/en-us/arcgis/products/arcgis-pro/overview)).

Shandong Province is located in the eastern part of China. It has a warm, temperate monsoon climate. Except for the east coast of the Jiaodong Peninsula, the continental climate is significant. It is affected by the combined effects of atmospheric circulation, monsoon, and topographic conditions, and the interannual variation of precipitation. The summer precipitation accounts for 60–70% of the annual rainfall. Small and medium-sized rivers are densely distributed in the province, including Tuhaimajia River system, Huayuankou River system, Yishusi River system, Shandong Peninsula coastal river system and so on. The spatial distribution of annual runoff in Shandong Province is uneven^24^., and the distribution during the year is also uneven. The runoff in flood season, especially in July and August, accounts for about 80% of the annual natural runoff. The study area of Shandong Province is shown in Fig. 1.

### GRACE data

The spherical harmonic product from GRACE and GRACE-FO with Level-2 RL06.1 version is used in this paper, provided by the Center for Space Research (CSR) of the University of Texas in the United States from January 2003 to December 2022. The data product after decorrelation and denoising kernel 3 (DDK3) filtering is adopted to filter the solid noise for extracting the terrestrial water storage (TWS) change. The first-order data of GSM should be added by the results from the TN13 file^25^. C_20_ and C_30_ value is substituted with corresponding C_20_ and C_30_ in the TN14 file from CSR^26,27^. The missing data in gap period of GRACE and GRACE-FO is compensated by TWS changes released by Zhong et al.^28^ in the Qinghai-Tibet Plateau Data Science Center. Missing data for individual months could be obtained by SSA interpolation.

### GLDAS model

The GLDAS global land surface model version 2.1 with the spatial resolution 0.25° × 0.25° and monthly time resolution, established by USA, Goddard Earth Sciences Data and Information Services Center (GES DISC), was adopted with the same time span as the GRACE data. The model reflects the monthly parameters changes such as soil water, ice and snow, and vegetation water content on the earth’s surface (https://mirador.gsfc.nasa.gov/). There are four models, which are NOAH, VIC, CLM and MOSAIC model. Meanwhile, GLDAS grid data is spherically expanded to the same order as GRACE data. In addition, the soil water, ice and snow, and vegetation water content changes in the study area are estimated by removing the average, combined filtering, and scale factor recovery methods. To reduce the uncertainty from each hydrological model, the mean values of four models for GLDAS are taken here.

### Groundwater data from WGHM

The surface water and groundwater data used in this paper are from the Watergap Global Hydrological Model (WGHM), which belongs to the monthly global terrestrial hydrological model (2.2c), including surface water and groundwater^29^. The model has been developed by the Institute of Physical Geography (IPG, University of Frankfurt, Germany). Its spatial resolution is 0.5° × 0.5°, one value per month, and the unit is mm. The model is constructed by monthly time series parameters, climate, and geographic data. The time span of the WGHM data ranges from 2003 to 2019, obtained by applying to the Müller S H professor from IPG, Germany^29^. The Groundwater hydrological data from WGHM is adopted in this paper to compare with the results from GRACE.

### GPCP precipitation model

The monthly GPCP data is derived from the Global Precipitation Climatology Centre (GPCC). The data is constructed by integrating the microwave and infrared data from dozens of geostationary satellites and polar-orbiting satellites, which is corrected by the data from multiple measured stations worldwide to obtain global satellite precipitation products. In this paper, the monthly data with the 2020 version is selected from January 2003 to April 2021, which can be downloaded from the website: https://www.psl.noaa.gov/data/gridded/data.gpcc.html. The spatial latitude and longitude coverage is 90° S ~ 90° N, 0° E ~ 360° E, its resolution is 1° × 1°.

### In-situ groundwater volume and level data

In this paper, the inversion results from GRACE data are comprehensively compared with in-situ data of annual precipitation, groundwater level, the volume of groundwater resources, groundwater mining, and water consumption for agricultural irrigation in Shandong Province from 2003 to 2021^30^. The data unit of precipitation is mm, and the latter two are km^3^. The data is from water resources bulletin issued by Shandong Provincial Department of Water Resources (http://wr.shandong.gov.cn/zwgk_319/fdzdgknr/tjsj/).

### Gap data compensation for GRACE and GRACE-FO

Given missing gap data from two generations of gravity satellites, a set of TWS change data based on precipitation reconstruction in China (2002.04–2019.12) is adopted in this paper, which has been released by the National Qinghai-Tibet Plateau Scientific Data Center^31,32^. High-precision China’s gridded gauge-based Daily Precipitation Analysis (CGDPA) precipitation products and CN05.1 temperature products in China are used in the data. The data gap between GRACE and GRACE-FO satellites for more than one year could be supplemented by this dataset.

## Methods

### Inversion method on GWS change

GRACE data are used to invert the TWS change in the study area, the formula of which can be expressed based on the change of equivalent water height $\Delta h_{w} (\varphi ,\lambda )$.

1 $$\Delta h_{w} (\varphi ,\lambda ) = \frac{{R\rho_{e} }}{{3\rho_{w} }}\sum\limits_{n = 0}^{N} {\sum\limits_{m = 0}^{n} {\frac{2n + 1}{{1 + k_{n} }}[\Delta \hat{C}_{nm} \cos m\lambda + \Delta \hat{S}_{nm} \sin m\lambda ]\overline{P}_{nm} (\cos \theta )} }$$

where $(\phi ,\lambda )$ are the geocentric colatitude and longitude of ground points;$(\Delta C_{nm} ,\Delta S_{nm} )$ are filtered potential coefficient with degree *n* and order* m*; $\overline{P}_{nm} ( \cdot )$ is the normalized associative Legendre functions; $k_{n}$ is the load LOVE number with degree* n* ; $\rho_{w} \approx 10^{3} kg/m^{3}$ is the density of water; $\rho_{e} \approx 5.5 \times 10^{3} kg/m^{3}$ is the average density of solid earth; *R* is the radius of the Earth.

The TWS change (∆*TWS*) actually includes the sum of soil water (*SMS*), ice and snow content (*SnWS*), surface water storage (*SWS*) represented by rivers, lakes and reservoirs, the vegetation water content (*CWS*), and GWS. It can be expressed by the following formula:

2 $$\Delta TWS = \Delta SnWS + \Delta SWS + \Delta CWS + \Delta SMS + \Delta GWS$$

The $\Delta TWS$ could be obtained from GRACE data. Because the GLDAS model is widely used to estimate the *SnWS* and *SWS*^4–6^*.* Therefore, the GLDAS with 2.1 version hydrological model of the same period can be adopted to obtain $\Delta SnWS$, $\Delta SMS$, $\Delta CWS$. WGHM model is used to obtain $\Delta SWS$. ΔGWS, the change of GWS, can be estimated from the formula $\Delta GWS = \Delta TWS - \Delta SnWS - \Delta SWS - \Delta CWS - \Delta SMS$.

### SVM method

Let the given nonlinear training set sample be $S{ = }\left\{ {\left( {x_{i} ,y_{i} } \right),\;\;i = 1,2, \ldots ,n} \right\}$, $x_{i} \in S^{n}$. Find a nonlinear function $\varphi \left( \cdot \right)$ and construct a linear optimal classification hyperplane $f\left( x \right) = w\varphi \left( \cdot \right) + b$, where the vector $w \in S^{n}$, $b \in S^{1}$, $f\left( x \right)$ is the output value of model. In order to solve the regression fitting problem using SVM, the specific problem can be described as an error function model :

3 $$\frac{\lambda }{2}\left\| w \right\|^{2} + \frac{1}{2}\sum\limits_{i = 1}^{n} {\left( {y_{i} - f\left( {x_{i} } \right)} \right)}^{2}$$

On the basis of classification, the error function is introduced to find the absolute maximum $\varepsilon$ of the sum of the model output value and the real output value. The quadratic error function is replaced by the insensitive error function $E_{\varepsilon }$, and the error model can be described as:

4 $$\frac{1}{2}\left\| w \right\|^{2} + C\sum\limits_{i = 1}^{n} {E_{\varepsilon } \left( {y_{i} - f\left( {x_{i} } \right)} \right)}^{2}$$

where, *C* is the penalty coefficient; $E_{\varepsilon }$ is an insensitive loss function.

5 $$E_{\varepsilon } \left( {y_{i} - f\left( {x_{i} } \right)} \right) = \left\{ {\begin{array}{*{20}l} {0,} \hfill & {\left| {y_{i} - f\left( {x_{i} } \right)} \right| \le \varepsilon } \hfill \\ {\left| {y_{i} - f\left( {x_{i} } \right)} \right| - \varepsilon ,} \hfill & {other\;case} \hfill \\ \end{array} } \right.$$

On this basis, two slack variables $\xi$, $\xi^{*}$ are introduced, then SVM optimization problem can be written as:

6 $$\begin{aligned} & \min \frac{1}{2}\left\| w \right\|^{2} + C\sum\limits_{i = 1}^{n} {\left( {\xi_{i} { + }\xi_{i}^{*} } \right)} \\ & \quad \;\;s.t.\;\;y_{i} \le f\left( {x_{i} } \right) + \varepsilon + \xi_{i} ; \\ & \quad \;\;y_{i} \ge f\left( {x_{i} } \right) - \varepsilon - \xi_{i}^{*} ;\;\;\;\xi_{i} \ge 0\;\;\;\;\xi_{i}^{*} \ge 0\;\;\;\;i = 1 \ldots n. \\ \end{aligned}$$

According to the Karush-Kuhn-Tucher (KKT) condition, the vector product of the Lagrange multiplier and constraint condition at the optimal point is 0. Then, it can be solved, parameter *b* is:

7 $$b = f\left( {x_{i} } \right) - \varepsilon - \sum\limits_{j = 1}^{n} {\left( {a_{j} { - }a_{j}^{*} } \right)} K\left( {x_{i} ,x_{j} } \right)$$

The prediction function is:

8 $$f\left( x \right) = \sum\limits_{i = 1}^{n} {\left( {a_{i} { - }a_{i}^{*} } \right)} K\left( {x_{i} ,x} \right) + f\left( {x_{j} } \right) - \varepsilon - \sum\limits_{i = 1}^{n} {\left( {a_{i} { - }a_{i}^{*} } \right)} K\left( {x_{i} ,x_{j} } \right)$$

**Figure 2:**
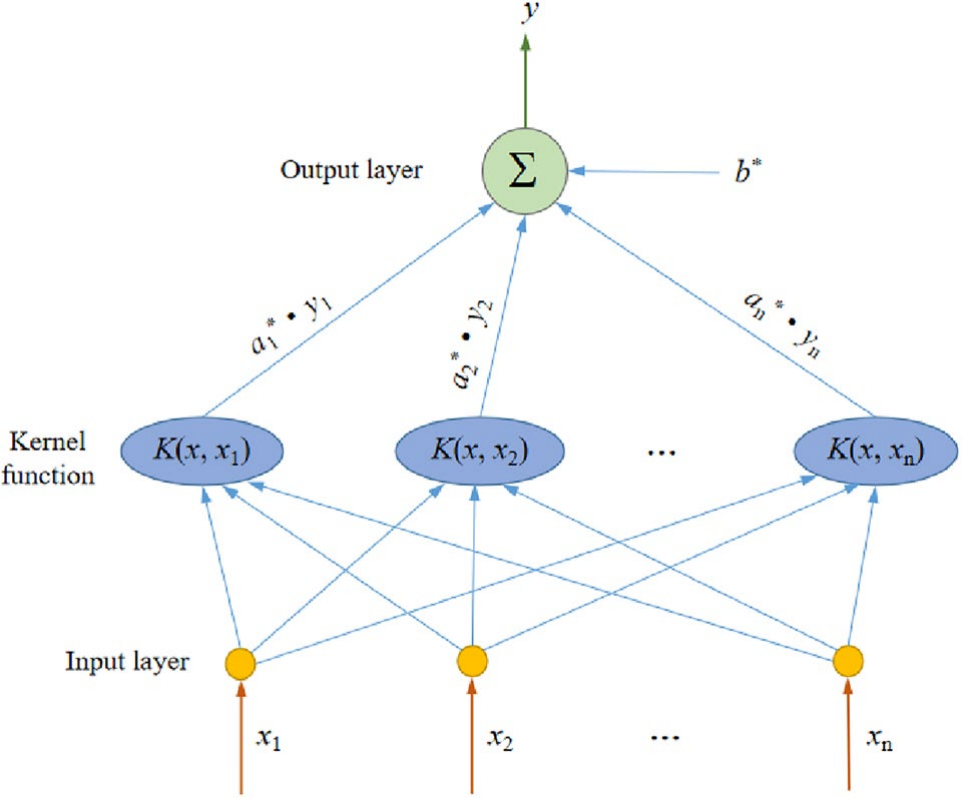
SVM network structure diagram.

The SVM network structure method is used in this paper. The structure diagram is shown in Fig. 2, $x_{i} \left( {0 < i < n} \right)$ is the input vector, $K\left( {x,x^{\prime}} \right)$ is the vector mapped from the low-dimensional space to the high-dimensional space by the kernel function, $a_{i} \sim a_{i}^{*}$ is the weight of network. $f\left( x \right)$ is the output value of the SVM network.

**Figure 3:**
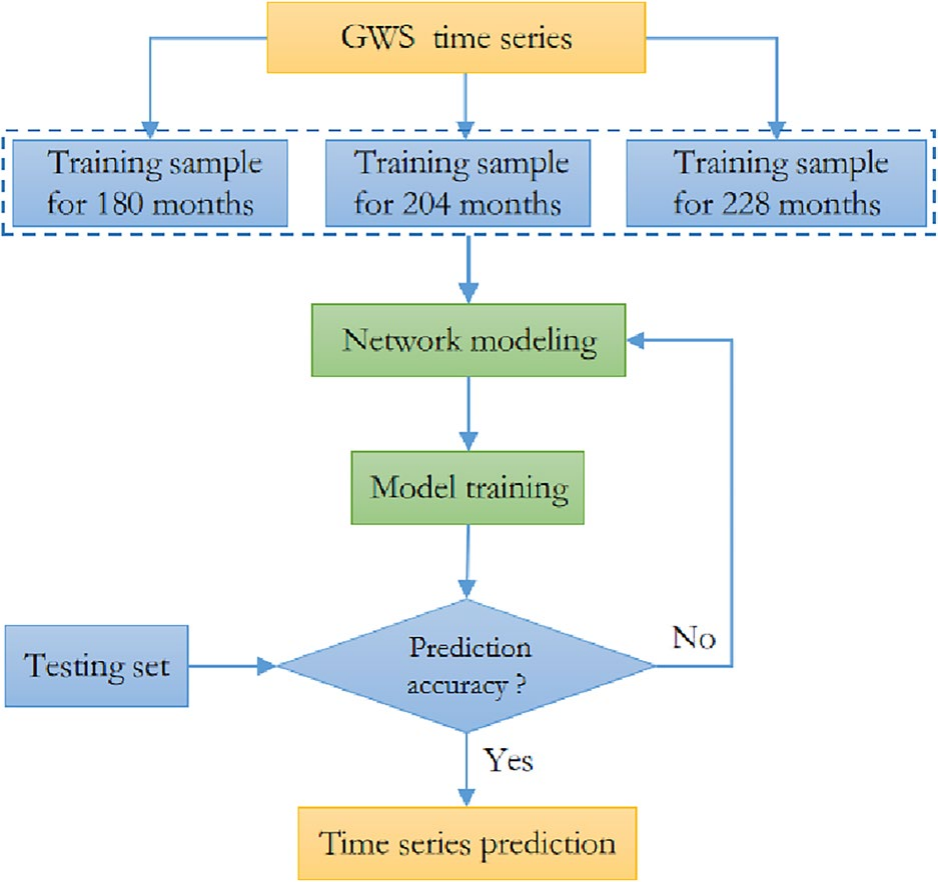
Flow chart of deep learning prediction with SVM method.

Based on the basic principle of SVM, the time series $(t_{i} ,y_{i} ),\;\;\;i = 1,2, \ldots ,240$ of GWS change from GRACE is analyzed in this paper. The prediction accuracy is compared with that from LSTM, SSA and ARMA methods. Taking *n* consecutive data of GWS change as training samples, the optimal model is established through multiple training and learning, GWS change with short-term, medium-term, long-term in the *n* + 12, *n* + 36 and *n* + 60 months is predicted, respectively. The prediction process is shown in Fig. 3.

Taking the time series of GWS change derived from GRACE as an example, the short-medium-long-term training of prediction was carried out with different samples of 228, 204 and 180 months, respectively, so the SVM network model was established. The initial setting of SVM model is that the gradient descent method is used to find the optimal penalty parameter *C*, the kernel function parameter, the loss function parameter is 0.01. The training model established depending on SVM method is used to predict the change of GWS on the different time-scale.

### Evaluation index of prediction accuracy

The correlation coefficient *r*, *NSE* (Nash–Sutcliffe Efficiency), root mean square error (*RMSE*) and sample determination coefficient (*R*^2^) are used to comprehensively evaluate the accuracy of SVM prediction method in this paper. The calculation formulas are as follows:

9 $$r\left( {x,y} \right) = \frac{{\sum\nolimits_{i = 1}^{n} {\left( {x_{i} \frac{{}}{{}}\overline{{x_{i} }} } \right)\left( {y_{j} \frac{{}}{{}}\overline{{y_{j} }} } \right)} }}{{\sqrt {\sum\nolimits_{i = 1}^{n} {\left( {x_{i} \frac{{}}{{}}\overline{{x_{i} }} } \right)^{2} \sum\limits_{i = 1}^{n} {\left( {y_{j} \frac{{}}{{}}\overline{{y_{j} }} } \right)^{2} } } } }}$$

10 $$NSE = 1{ - }\frac{{\sum\nolimits_{i = 1}^{n} {\left( {x_{i} - \hat{x}_{i} } \right)^{2} } }}{{\sum\nolimits_{i = 1}^{n} {\left( {x_{i} - \overline{x}} \right)^{2} } }}$$

11 $$RMSE = \sqrt {\frac{{\sum\nolimits_{i = 1}^{i = n} {\left( {x_{i} - \hat{x}_{i} } \right)^{2} } }}{n}}$$

12 $$R^{2} { = 1} - \frac{{\sum\nolimits_{i = 1}^{n} {\left( {y_{i} - \hat{y}_{i} } \right)^{2} } }}{{\sum\nolimits_{i = 1}^{n} {\left( {y_{i} - \overline{y}} \right)^{2} } }}$$

Among them, *n* represents the number of samples, that is, different prediction durations, $i\left( {i{ = }1,2,3 \ldots ,n} \right)$ is each monthly time point, $\hat{x}_{i}$ is the true value of the predicted signal, $\overline{x}$ is the average value of the predicted signal, $x_{i}$ representing the actual predicted value. $y_{i}$ is the prediction data, $\hat{y}_{i}$ is the actual data, *n* is the number of samples. *R*^2^ represents the “goodness of fit” between the predicted value and the sample observation value. It should be noted that *R*^2^ is equal to 1, indicating that the predicted value and the true value of sample are equal, without any prediction error. The *NSE* range is from − ∞ to 1, and the closer the value is to 1, the better the consistency between the predicted time series and the actual amplitude signal.

## Results and analysis

### Spatio-temporal analysis on GWS fluctuation

**Figure 4:**
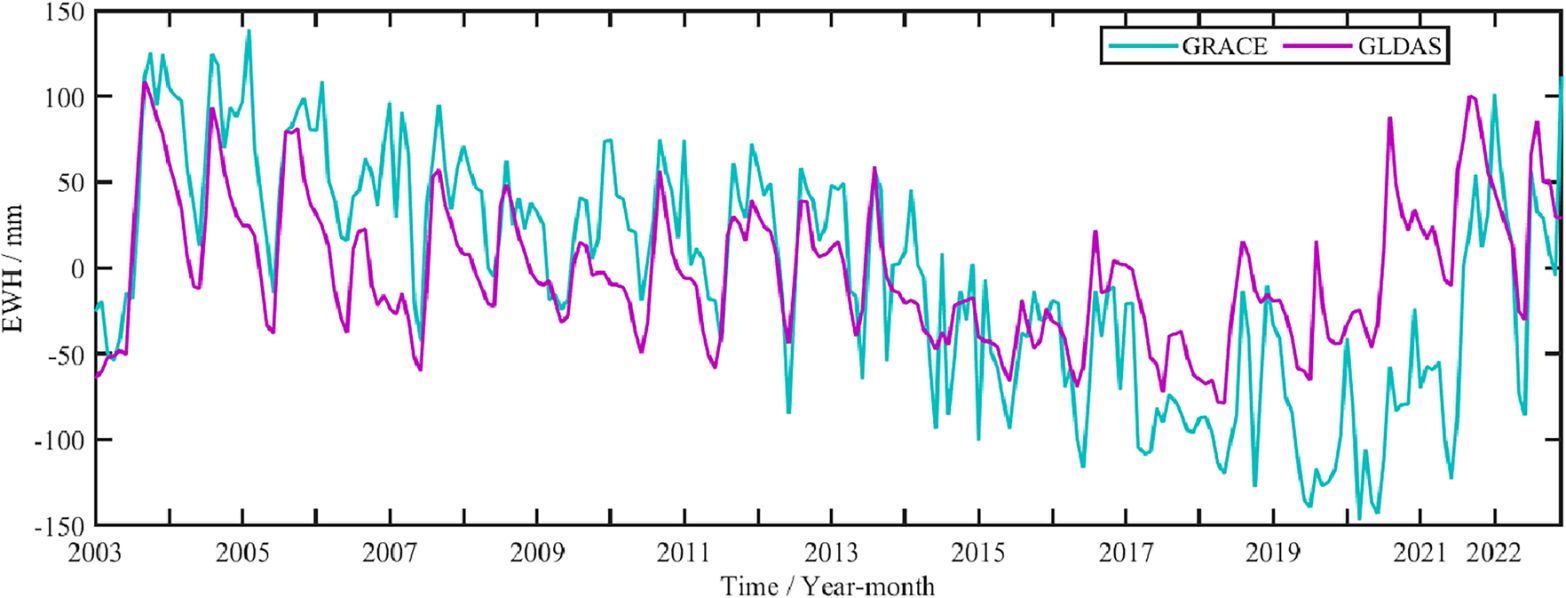
The comparison with the results from GLDAS and TWS from GRACE data.

In this paper, the DDK3 filtered product from CSR is used to obtain the change of TWS in Shandong Province from 2003 to 2022, the product has been filtered by CSR. In addition, the missing data in individual months rather than gap period are compensated by SSA method. Due to the gap from GRACE and GRACE-FO, the data is divided two parts, which is from January 2003 to June 2017, from June 2018 to December 2022, respectively. In addition, we compared the GLDAS data model with the TWS change from GRACE to ensure accuracy in the data obtained, shown in Fig. 4. The correlation coefficient between the results from GRACE and GLDAS is 0.65, which has a good correlation.

**Figure 5:**
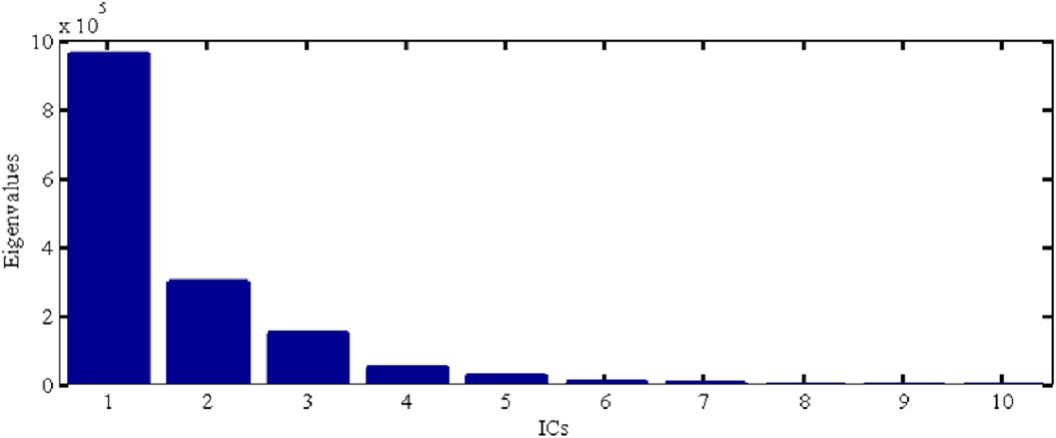
The first eight principal component eigenvalues of GWS change in Shandong decomposed by ICA.

Furthermore, depending on the GLDAS and WGHM model, GWS change in Shandong Province is obtained according to formula (2). To reveal the more detailed characteristics of GWS changes, the ICA method is used in this paper to decompose the time series signal of GWS change from January 2003 to June 2017 derived from GRACE. The time series signal is decomposed into several independent components IC1, IC2, IC3 and IC4, which reflect the characteristics of different periods and trend terms. Meanwhile, the data has been centralized, and how to select the number of principal components is the critical link of the ICA method to extract signals. Firstly, the self-covariance matrix is constructed based on the principle of the ICA method by using the centralized data. Secondly, the constructed autocovariance matrix is diagonalized. Thirdly, the eigenvalues obtained by the eigenvalue decomposition method are sorted from large to small, as shown in Fig. 5. Finally, the number of spatio-temporal pattern components is selected according to the percentage of each principal component in the total energy. The size of the eigenvalue represents the contribution of the corresponding eigenvector to the entire matrix after the matrix is orthogonalized.

Figure 5 shows that the first characteristic value accounts for the highest proportion. The first four independent component signals already contain the primary information of regional GWS change of about 97.5%, which can fully explain the change of GWS. The component signal after the fifth is relatively small and could not be considered. Therefore, the first four components from spatio-temporal change of GWS in Shandong Province may be analyzed in this paper.

**Figure 6:**
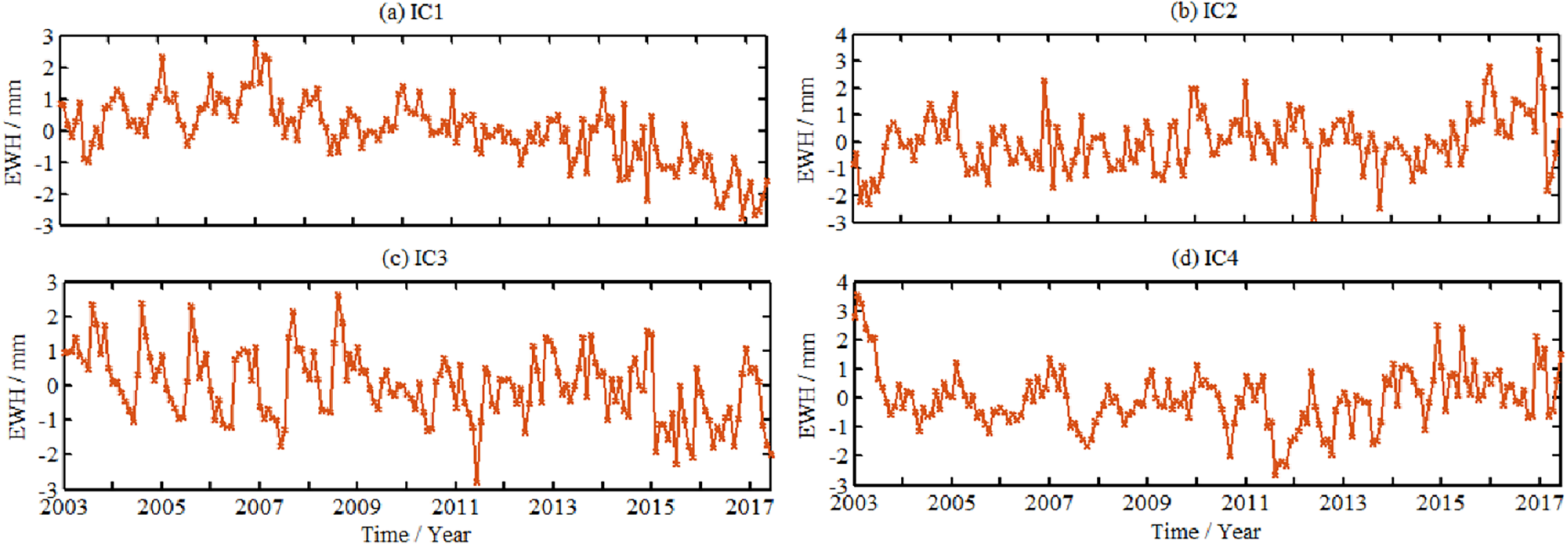
The time principal component of GWS change decomposed by ICA in Shandong Province (It was generated by GMT software (https://gmt-china.org)).

**Figure 7:**
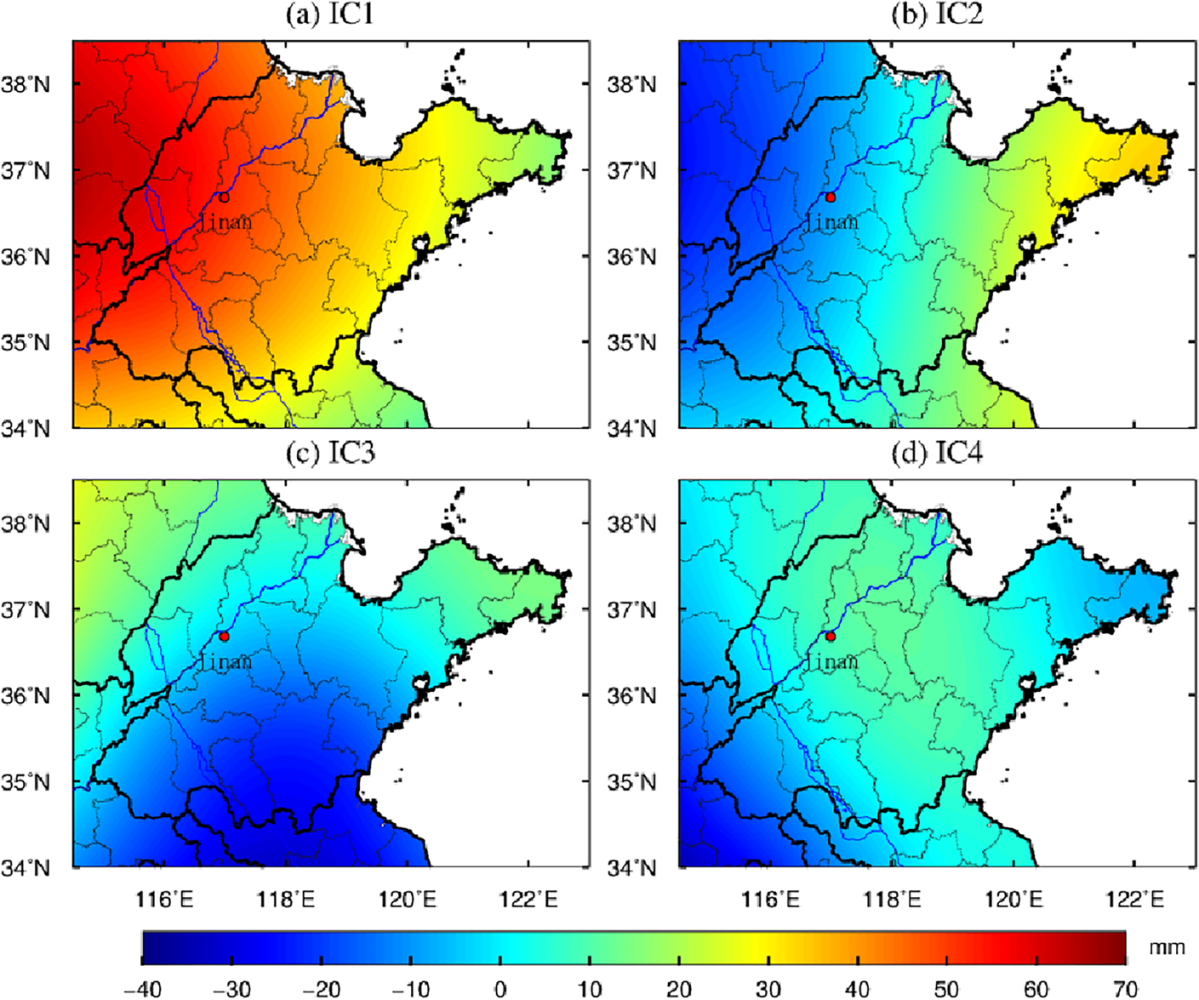
The spatial principal component of GWS change decomposed by ICA in Shandong Province (It was generated by GMT software (https://gmt-china.org)).

The spatio-temporal features extracted by ICA method are shown in Figs. 6 and 7, respectively. Normalization processing has been performed, and the four principal components of spatio-temporal maps are arranged from large to small according to the proportion of eigenvalues. The unit of time pattern diagram in Fig. 4 is dimensionless, there is no actual physical meaning, but only the numerical size is represented. The numerical unit of spatial pattern in Fig. 7 is mm. The colors of spatial signal in Fig. 7 have positive and negative value. The value greater than zero indicates that the change of GWS in Shandong Province is the same as corresponding component in Fig. 6. Values less than zero indicate the opposite trend. The actual change of each component of GWS needs to be multiplied by corresponding temporal results from Fig. 6.

It can be seen from Fig. 6 that during the period from January 2003 to June 2017, the GWS in Shandong Province decomposed by ICA method were dominated by the changes with long-term trend. To more clearly distinguish the periodic characteristics of each principal component, the fast Fourier transform algorithm (FFT) is adopted to detect the first four principal components. The results show that IC1 component has obvious trend changes. The principal components of the IC2 and IC3 signal showed a monthly periodic signal. The IC4 signal has a significant piecewise linear trend, which decreased obviously from 2003 to 2011, showing an increasing trend after 2012.

Combined with Figs. 6 and 7, it can be found that GWS represented by IC1 shows that there is different trend between 2003–2007 and 2007–2017, showing a increasing trend and a decreasing trend continuously, respectively. This deficit signal is relatively strong in the western region in Shandong Province, the signal intensity shows a stepwise distribution from inland to coastal. The monthly cycle signals represented by the principal components of IC2, IC3 showed spatial inhomogeneity. IC4 indicates that GWS in the central region decreased continuously during 2003, it is relatively stable between 2004 and 2011 year although fluctuates. It indicates that GWS showed a significant downward trend from 2007 to June 2017, and the intensity of GWS loss in the western area was significantly greater than that the coastal region.

**Figure 8:**
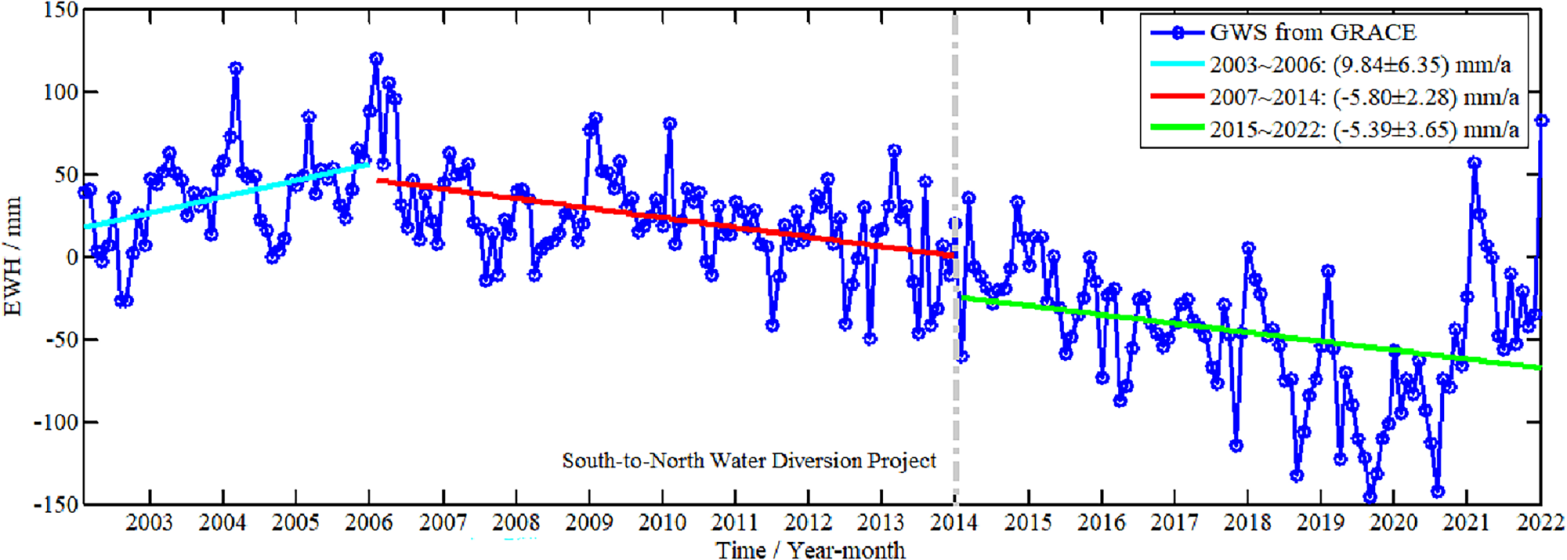
Variation of GWS and its linear trend in Shandong Province during the recent 20 years.

On this basis, the data products released by Yulong Zhong is adopted to fill the gap missing data for GRACE and GRACE-FO, so continuous time series of TWS changes from January 2003 to December 2022 is obtained, as shown in Fig. 8, a total of 240 months. There are distinctions between Figs. 5 and 7. Figure 5 shows the time changes of the four principal components, with high energy proportion after the ICA decomposition of GWS in Shandong Province. Figure 7 shows the overall time series of GWS changes in Shandong Province.

Considering the impact of the East Route of the South-to-North Water Diversion Project on the change of GWS in Shandong Province since its implementation in 2014, the three different linear trends of GWS changes are obtained in GRACE inversion from 2003 to 2006, 2007 to 2014 and 2015 to 2022, respectively, which are shown as cyan, red, and green curve. From Fig. 8, we can find that the linear change rate of GWS from 2003 to 2006 is 9.84 ± 6.35 mm/a; there is an apparent loss in the GWS from GRACE, its linear rate from 2007 to 2014 is − 5.80 ± 2.28 mm/a; the trend of GWS from GRACE loss weakened, linear rate from 2015 to 2022 is − 5.39 ± 3.65 mm/a, which may be owing to the effect of South-to-North Water Diversion Project. However, after 2014, the loss trend of GWS from GRACE continued to exist until 2019. From 2020 to 2021, GWS rebounded sharply, and the specific reasons need to further study.

### Comparison with model data

#### Comparison with WGHM model

**Figure 9:**
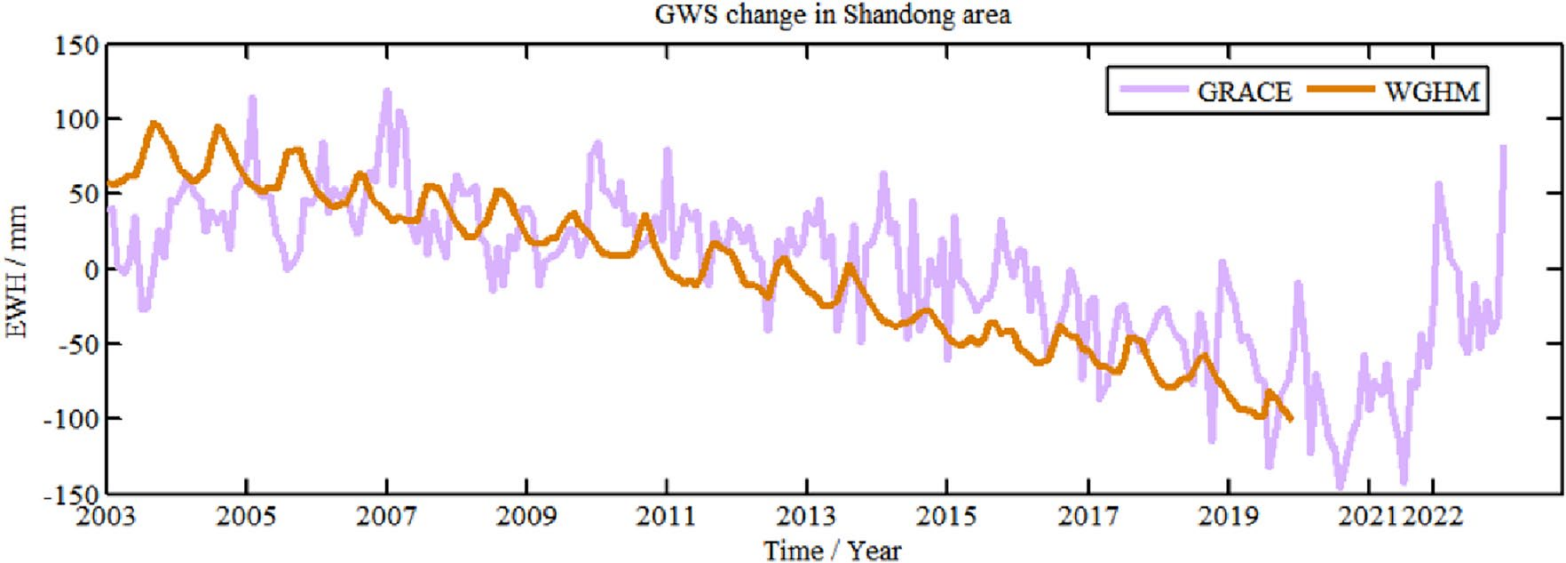
Comparison between GWS change in Shandong Province from GRACE and WGHM.

To verify the validity of GWS change derived from GRACE, WGHM model is used for comparative analysis, as shown in Fig. 9, which are averaged for region. Since the current WGHM is only updated to December 2019, the brown curve in Fig. 9 represents GWS change from WGHM model from January 2003 to December 2019.

It can be found from Fig. 9 that GWS from GRACE obviously increases between 2003 and 2006, GWS from GRACE obviously decreases from 2004 to 2019. However, overall, there is decreasing trend of GWS derived from GRACE between 2003 and 2019, which is consistent with global groundwater hydrological model from WGHM. In addition, the differences between the two results during 2003 to 2006 means that there are some uncertainties in the results from GRACE and WGHM model. The correlation coefficient of both the results is 0.67, which is positively correlated, indicating that WGHM model can better verify GRACE results.

#### Comparison with monthly precipitation from GPCP

**Figure 10:**
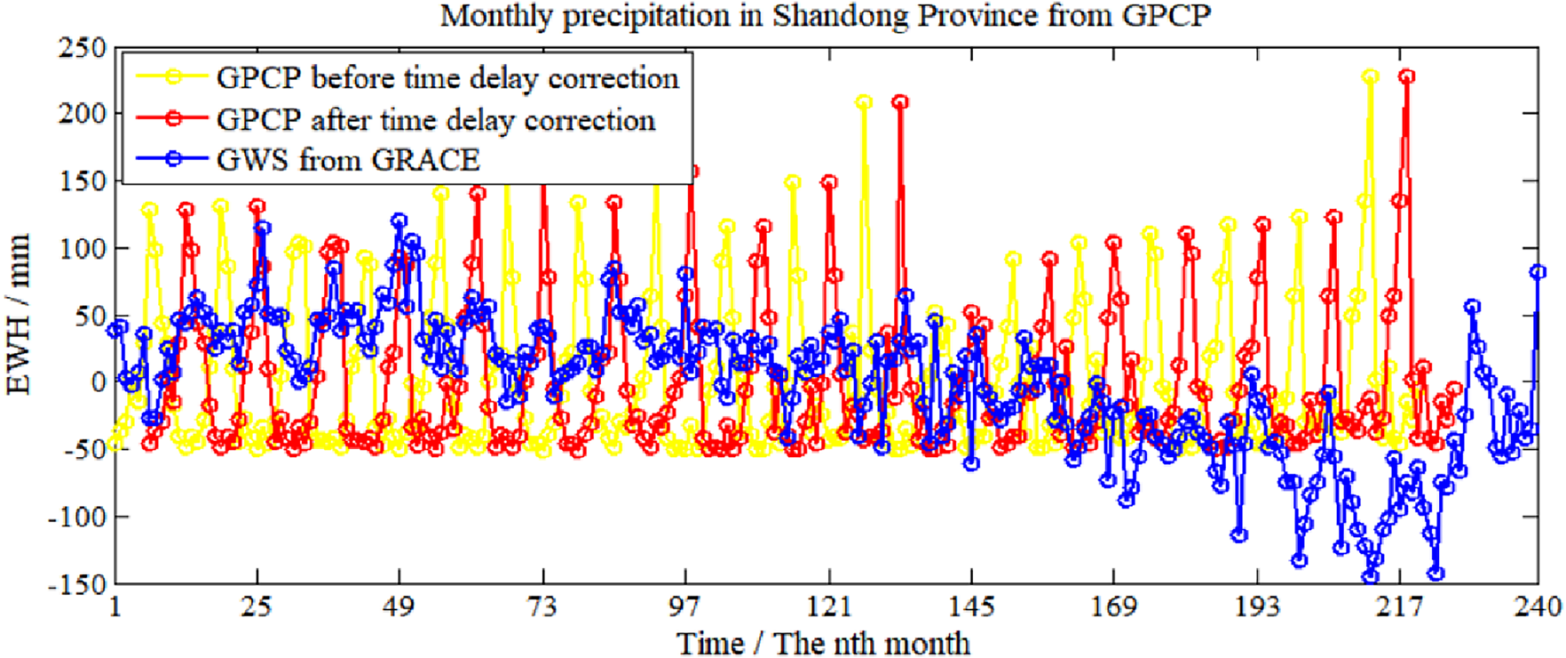
Comparison between GWS change in Shandong Province from GRACE and GPCP.

To analyze the relationship between the change of GWS and precipitation, the GPCP model is used in this paper to obtain the monthly precipitation data in Shandong Province from January 2003 to April 2021, a total of 220 months, as shown in Fig. 10 with yellow curve. The red curve shows the monthly precipitation data after a time delay (6 months) correction. In addition, time delay corrections with different months for GRACE results are performed. The statistical results are shown in Table 1, *N* represents the month number of time delay.

**Table 1:** Correlation coefficient of monthly precipitation and the results from GRACE after time delay correction with different months.

Time delay	*N* = 0	*N* = 1	*N* = 2	*N* = 3	*N* = 4	*N* = 5	*N* = 6	*N* = 7	*N* = 8	*N* = 9	*N* = 10	*N* = 11	*N* = 12
Correlation coefficient	− 0.23	− 0.22	− 0.16	− 0.09	0.05	0.18	0.21	0.18	0.13	0.04	− 0.08	− 0.19	− 0.19

Significant values are in bold.

Combined with Fig. 10 and Table 1, it can be found that the correlation between the results from GRACE and the GPCP model is negatively correlated before correcting the time lag. When 0 < *N* < 6, the correlation coefficient is improved gradually. When *N* = 6, the positive correlation reaches the highest value of 0.21. When 6 < *N* ≤ 12, the correlation coefficient decreases, when *N* = 12, the negative correlation reaches the maximum. It indicates that precipitation is an essential factor of affecting the change of GWS in Shandong Province, from the perspective of hydrology, the infiltration process of precipitation from surface to underground is relatively slow, and it has an impact on GWS in Shandong Province with time delay (6 months).

**Figure 11:**
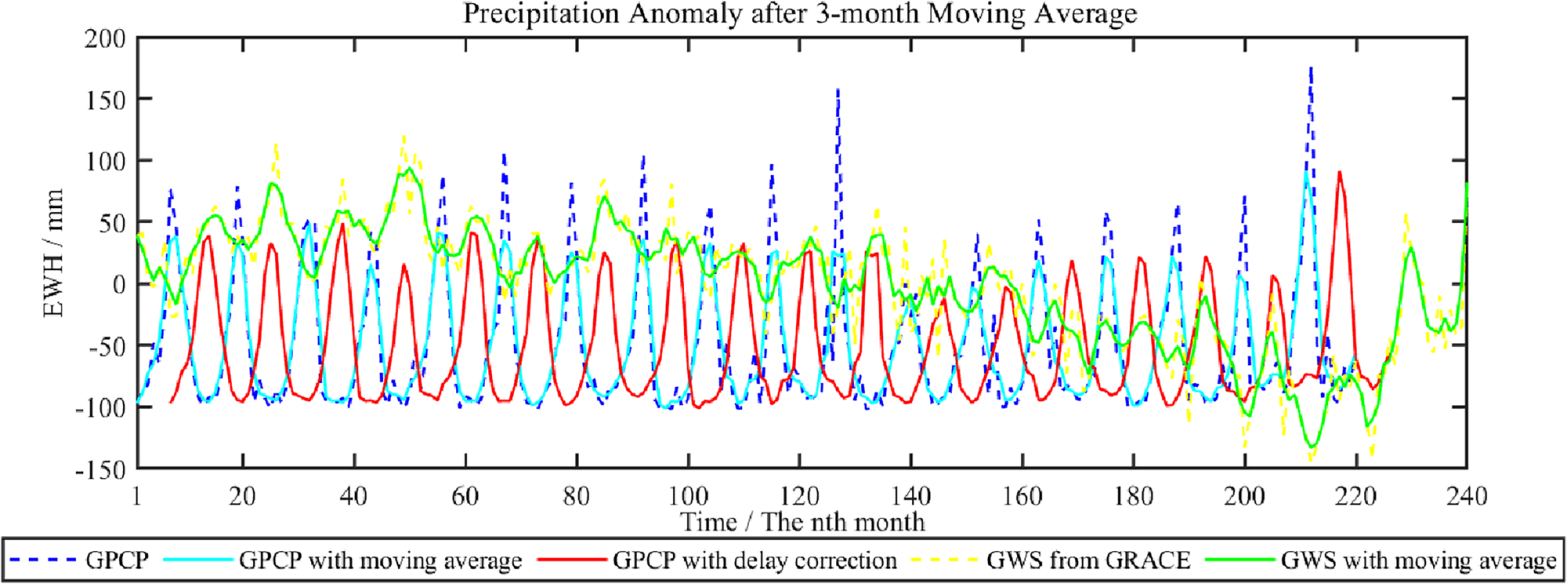
Comparison between GWS change in Shandong Province derived from GRACE and GPCP with a 3-month moving average.

To facilitate the comparison between GPCP data and GWS from GRACE, precipitation anomaly value of monthly time series could be calculated firstly, and then the 3-month moving average method is adopted to eliminate the random fluctuation of both the time series. In Fig. 11, cyan curve and green curve shows precipitation data and GWS from GRACE after moving average. In addition, the time delay correction with different months for precipitation data after the moving average is performed to determine the maximum correlation coefficient between precipitation data and GRACE results. The statistical results are shown in Table 2.

**Table 2:** Correlation coefficient of precipitation anomaly and GRACE after 3-month moving average with time delay correction of different months.

Time delay	*N* = 0	*N* = 1	*N* = 2	*N* = 3	*N* = 4	*N* = 5	*N* = 6	*N* = 7	*N* = 8	*N* = 9	*N* = 10	*N* = 11	*N* = 12
Correlation coefficient	− 0.27	− 0.26	− 0.19	− 0.08	0.05	0.17	0.23	0.21	0.14	0.03	− 0.09	− 0.19	− 0.23

From Table 2, when time delay correction *N* = 6, the positive correlation between precipitation anomaly and GRACE results after the 3-month moving average reaches the highest value of 0.23, shown as a red curve in Fig. 10, which improved to about 10% ((0.23–0.21)/0.21) comparing with the results without moving average.

**Figure 12:**
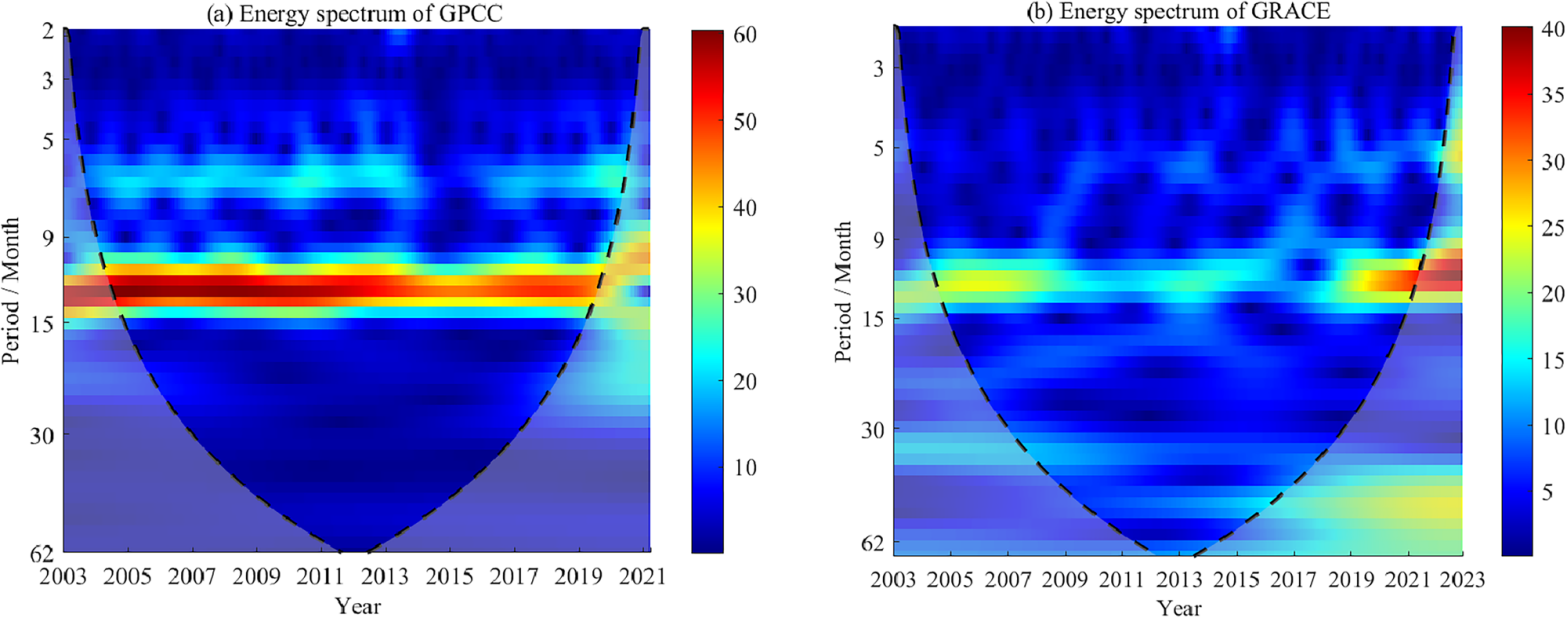
The energy spectrum of the time series from GRACE results and GPCP with the 3-month moving average in Shandong Province.

In addition, to further analyze the relationship between precipitation anomaly and GWS, the CWT method is used in this paper to obtain both the energy spectrum, which are shown as in Fig. 12a and b.

In Fig. 12, below the black dashed line is the wavelet influence cone area with significant data edge effect. We can find that monthly precipitation anomaly and GWS from GRACE after the 3-month moving average has the similar energy spectrum. There is obvious period signal with 12 months of both the data. However, during 2010 to 2017 year, seasonal signal is weaker in GRACE results than precipitation from GPCP model, indicating that human factors could has more significant effect than that of rainfall on GWS fluctuation from GRACE between 2010 and 2017.

### Comparison with actual observations

#### Comparison with in-situ groundwater data

**Figure 13:**
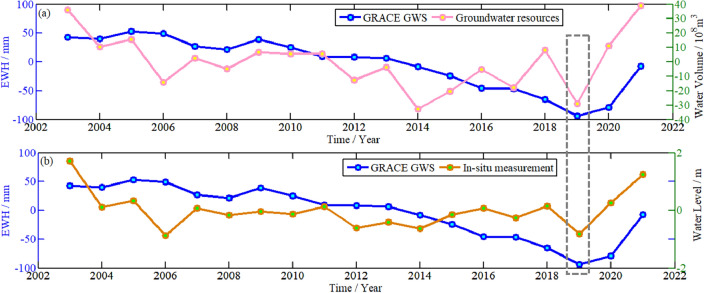
Comparison on the annual variation of GWS in Shandong Province from GRACE and in-situ data.

To further validate the results from GRACE, the in-situ data of GWS (unit: billion m^3^) is used in this paper, which has been released by the Shandong Provincial Department of Water Resources. These in-situ data is annual change, and groundwater level is shallow data. To ensure the unity of both the time resolution, the monthly variation of results derived from GRACE are processed by yearly average to obtain the annual change of GWS, which are shown with blue curve in Fig. 13. The red curve represents in-situ annual variation of groundwater resources, the yellow curve shows in-situ annual change of shallow groundwater level.

It can be seen from Fig. 13 that the results from GRACE are consistent with the in-situ annual variation of groundwater resources and groundwater level. From 2007 to 2019, the results from GRACE showed a decreasing trend continuously,both the data showed apparent losses in 2019. The GWS increased significantly from 2020 to 2021, in-situ data also showed a consistent trend. The annual GWS change from GRACE is positively correlated with the in-situ groundwater resources and groundwater level data, and the correlation coefficients between them are 0.34 and 0.18, respectively. It can be shown that GWS are consistent with the overall trend of in-situ data. However, there are still differences in detailed time characteristics, which may be owing to the low spatial resolution of GRACE, leading to some uncertainty in results.

#### Comparison with annual precipitation and human activities

To further analyze the factors influencing GWS changes in Shandong Province, the relevant data from the water resources bulletin issued by Shandong Provincial Department of Water Resources is adopted for comprehensive analysis, including annual rainfall, annual groundwater mining, and annual water consumption for agricultural irrigation.

**Figure 14:**
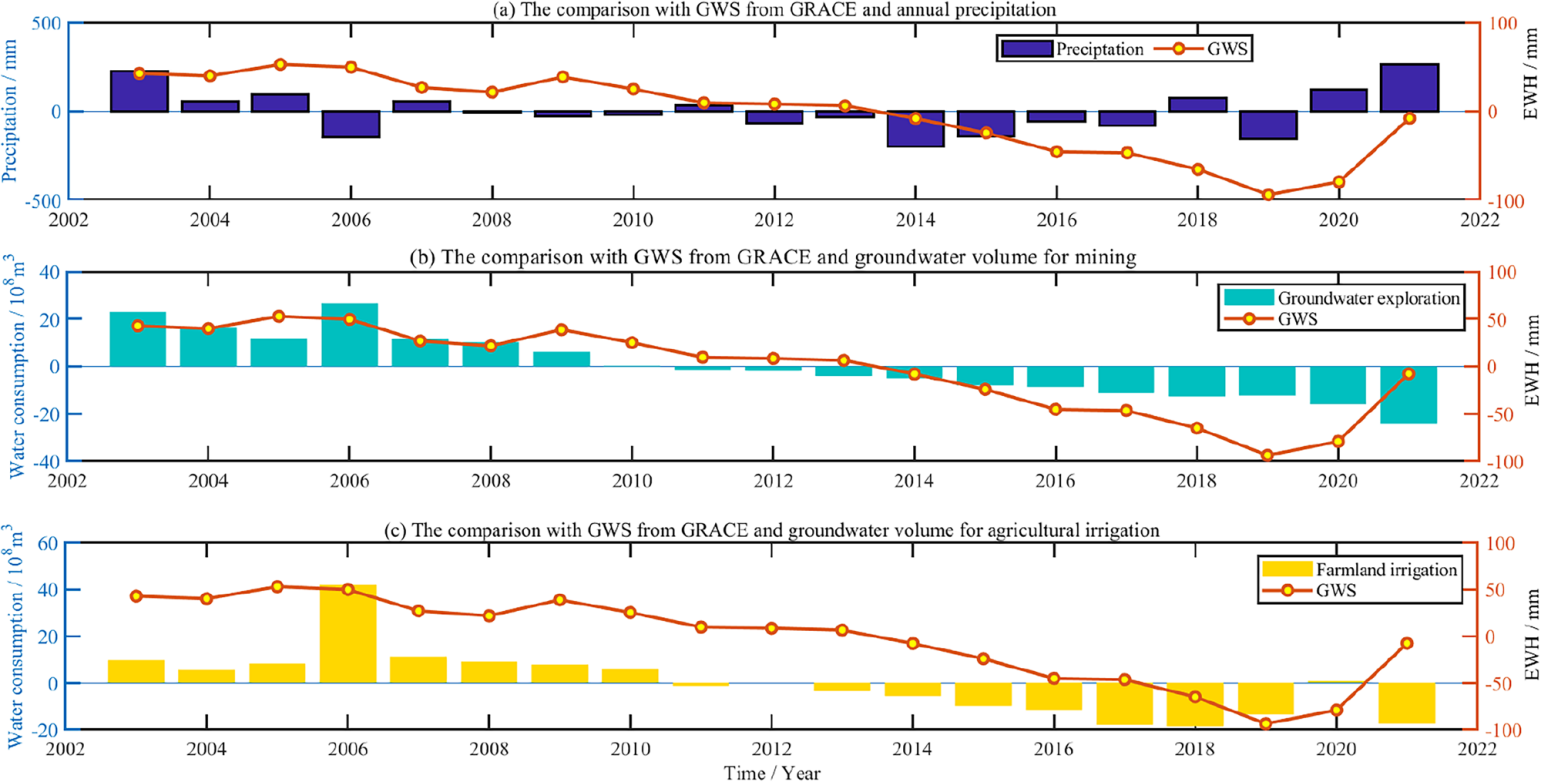
Comparison of the results derived from GRACE with annual precipitation anomaly and water consumption change caused by human factors.

To unify the time resolution for different data, the results derived from GRACE are averaged annually. Meanwhile, to unify the data of precipitation, groundwater mining and agricultural irrigation in the same time benchmark, the average value of every kind of data from 2003 to 2021 is deducted to reflect the annual anomaly value in this paper. In order to more intuitively analyze the impact of regional GWS change, the annual changes of GWS are compared with precipitation anomalies, annual groundwater mining, and water consumption for agricultural irrigation, as shown in Fig. 14a–c. The right axis in the three sub-diagrams represents the annual change of GWS, and the left axis represents the water volume change of three influencing factors. In addition, the water consumption for annual groundwater mining and agricultural irrigation in Fig. 14 has also been deducted from the average of all years.

From Fig. 14, it can be seen that annual variation of GWS in Shandong Province derived from GRACE is more consistent with the trend of annual groundwater exploration and farmland irrigation, on the whole. From 2009 to 2010, the GWS from GRACE showed a downward trend, while precipitation was less and the groundwater exploitation and farmland irrigation increased. During 2019, precipitation decreased severely, groundwater mining and farmland irrigation volume are all negative, GWS also decreased obviously. From 2020 to 2021, precipitation increased obviously, groundwater mining and farmland irrigation almost all decreased more remarkably, meanwhile, there is significant increase in GWS. In addition, the correlation coefficient is calculated between GWS and influencing factors. The correlation coefficient is 0.18, 0.80, 0.71, respectively, which are as follows in Table 3.

**Table 3:** The correlation coefficient between GWS from GRACE and precipitation, groundwater exploration, farmland irrigation.

Influence factors on GWS	Precipitation	Groundwater exploitation	Farmland irrigation
Correlation coefficient	0.18	0.80	0.71

### Prediction on GWS change

#### The predicted results from SVM

**Figure 15:**
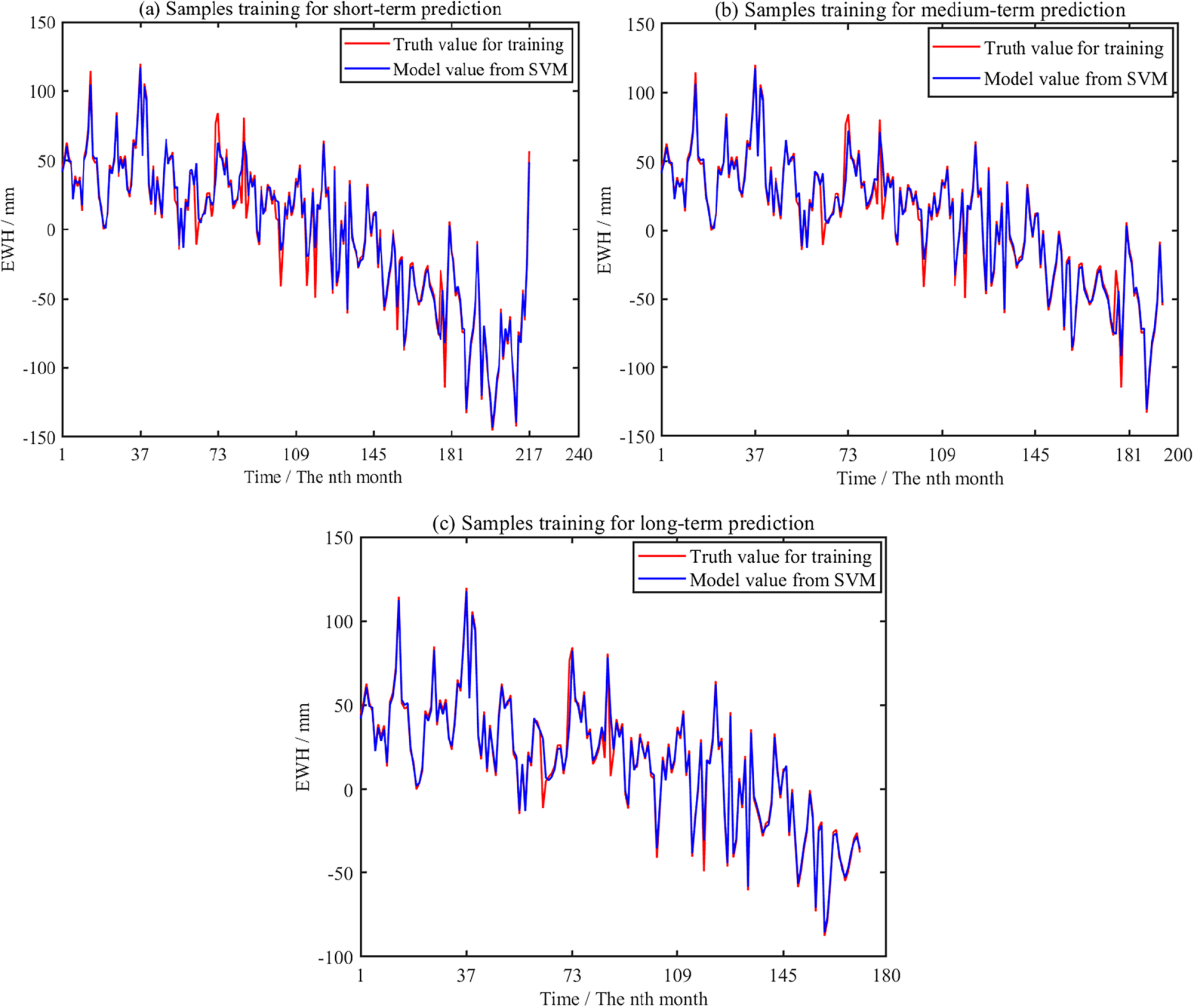
Short-medium-long term prediction for training samples of GWS change based on SVM method.

To predict the change of GWS in Shandong Province, based on the basic principle of SVM prediction, SVM method is adopted for predicting. The short-medium-long-term trend of predicting GWS change in Shandong Province is evaluated. In addition, SVM modeling is performed on the different training samples. The short-medium-long-term prediction training results are shown in Fig. 15, respectively.

Specifically, the time series of GWS change with 240 months is divided into training and testing samples, respectively. Taking the data with 228 months as training samples in short-term prediction, taking the data with 204 months as training samples in medium-term prediction, and taking the data with 180 months as training samples in long-term prediction.

The red curves in the Fig. 15a represent the original data of training samples, the blue curve shows the modeled value from SVM method in short-term prediction. The blue curves in the Fig. 15b and c shows the modeled value in medium-term and long-term prediction, respectively. The short-medium-long-term signal of prediction is very close to the original signal, and *R*^2^ is 0.97, 0.97 and 0.98, respectively.

**Figure 16:**
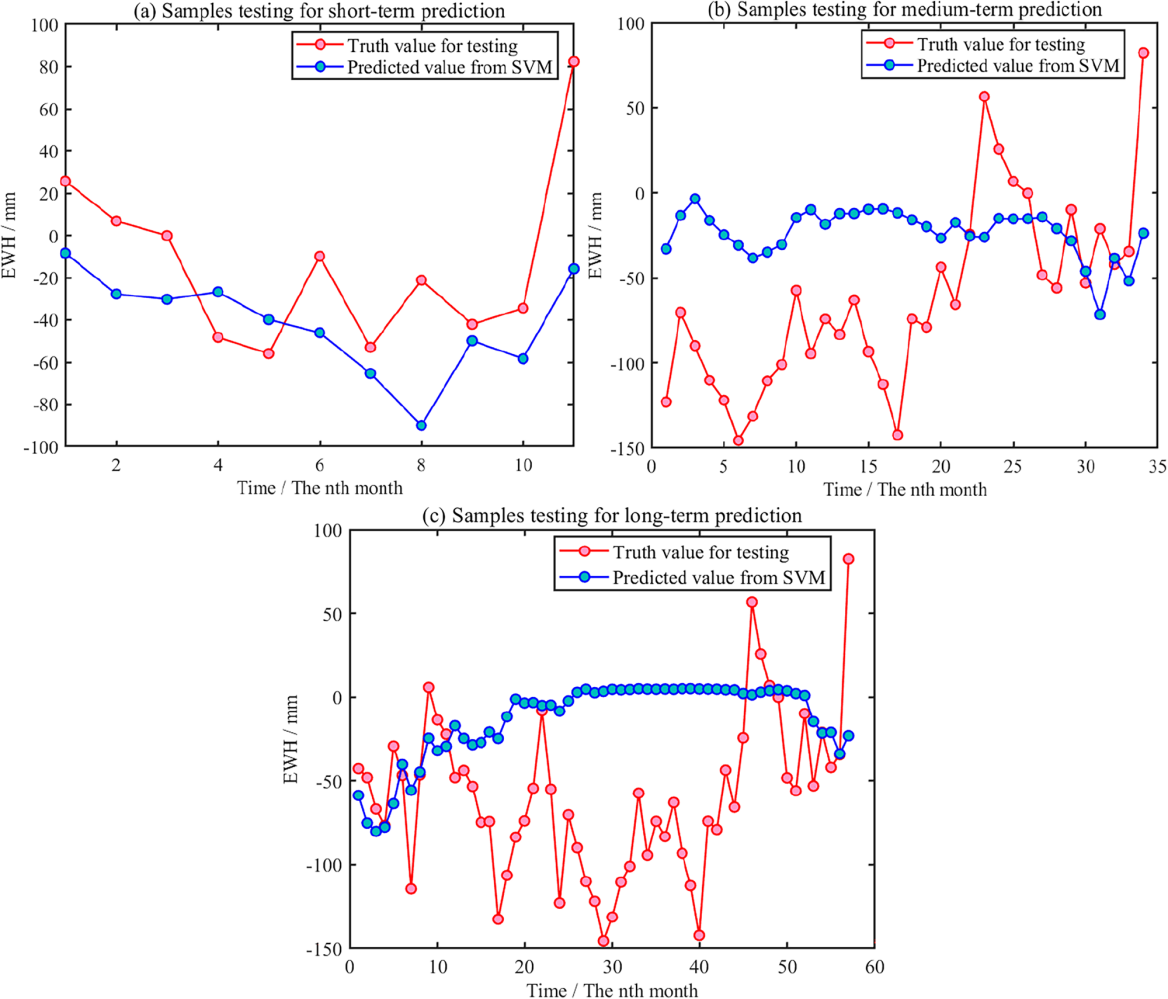
Short-medium-long term prediction for testing samples of GWS change from SVM.

Based on the above established SVM model, the time series of GWS in Shandong Province is predicted on the different time scale. The predicted results from SVM are compared with testing samples on short-medium-long terms, which are shown in Fig. 16. The evaluation indexes are *R*^2^, *RMSE,* and correlation coefficient. The statistical results are shown in Table 4.

**Table 4:** Evaluation for predicting GWS changes with different training samples using SVM method.

Training /testing precision evaluation	Short-term forecast (12 months)	Medium-term forecast (36 months)	Long-term forecast (60 months)
180 training samples (200,301–201,712)	*R*^2^ = 0.98/Correlation = 0.56	*R*^2^ = 0.98/Correlation = − 0.27	*R*^2^ = 0.98/Correlation = − 0.14
204 training samples (200,301–201,912)	*R *^2^= 0.97/Correlation = 0.75	*R*^2^ = 0.97/Correlation = − 0.06	
228 training samples (200,301–202,112)	*R*^2^ = 0.97/Correlation = 0.68		

It can be seen from Fig. 16 and Table 4 that based on different training samples, GWS change with short-term signal predicted by SVM method has a certain degree of agreement with the overall trend of testing sample. The time series of predicting GWS change with medium and long-term deviates significantly from the testing signal. The predicted time series is consistent with short-term testing samples, and the correlation coefficient is relatively high, reaching 0.65–0.75.

#### Comparison with other methods

**Figure 17:**
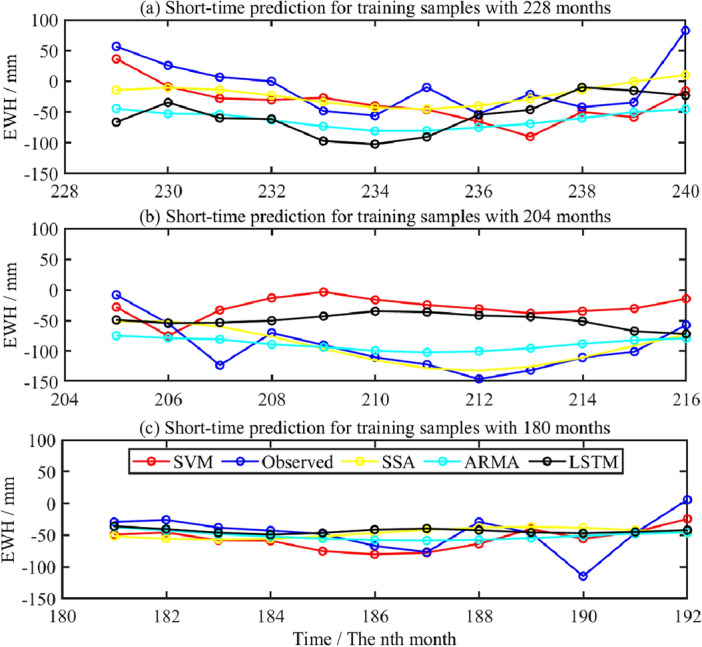
Prediction for GWS change in Shandong based on SVM and comparison with different methods.

To more effectively evaluate the superiority of predicting short-term changes of regional GWS depending on SVM method, the prediction results from SVM model is compared with that from SSA, ARMA and LSTM methods for short-term prediction. Figure 17a–c shows the predicted signal based on different training samples. The blue curve is regarded as an actual signal. The correlation coefficient, *NSE*, and *RMSE* are used to evaluate the prediction accuracy comprehensively.

It can be found from Fig. 17 that in terms of short-term prediction, the change of GWS predicted by SVM model is most consistent with the testing samples of GRACE. In addition, it is also consistent with the results from SSA and ARMA method, but it is quite different from that of LSTM method. To quantitatively evaluate the superiority of SVM method, the index of precision including correlation coefficient, *NSE*, *RMSE* are used, to comprehensively assess the predicted accuracy for the four methods in the short-term period. The predicted experiments with different training samples will be carried out, such as 228, 204, and 180 months. Table 5 shows the correlation coefficient, *RMSE* and *NSE* accuracy indexes of short-term prediction for GWS change with 228, 204 and 180 training samples, respectively.

**Table 5:** Comparison of short-term prediction accuracy of GWS change with different training samples.

Index of precision	SVM (228/204/180)	LSTM (228/204/180)	SSA (228/204/180)	ARMA (228/204/180)
Correlation coefficient	0.68/0.75/0.56/	0.26/− 0.39/0.26	0.65/0.79/− 0.38	0.72/ 0.73/0.44
*RMSE/*mm	5.65/4.42/5.26/	6.85/6.71/5.39	5.98/4.94/5.78	5.98/5.74/5.25
*NSE*	0.36/0.43/0.28/	− 0.12/− 0.30/0.05	0.35/0.62/− 0.26	0.35/0.30/0.15

It can be found from Table 5 that from the perspective of short-term prediction, the correlation coefficients between predicted results from the SVM model based on training samples of 228, 204, and 180 months and the accurate signals are 0.68, 0.75, and 0.56, respectively, which are significantly better than that from LSTM and SSA methods, but are not much different from the results of ARMA. *RMSE* were 5.65, 4.42, 5.26 mm, its accuracy index was better than that from the other three methods. The *NSE* values were 0.36, 0.43 and 0.28, respectively, which were superior to the other three methods. It shows that the SVM model has higher prediction accuracy, on the whole.

**Figure 18:**
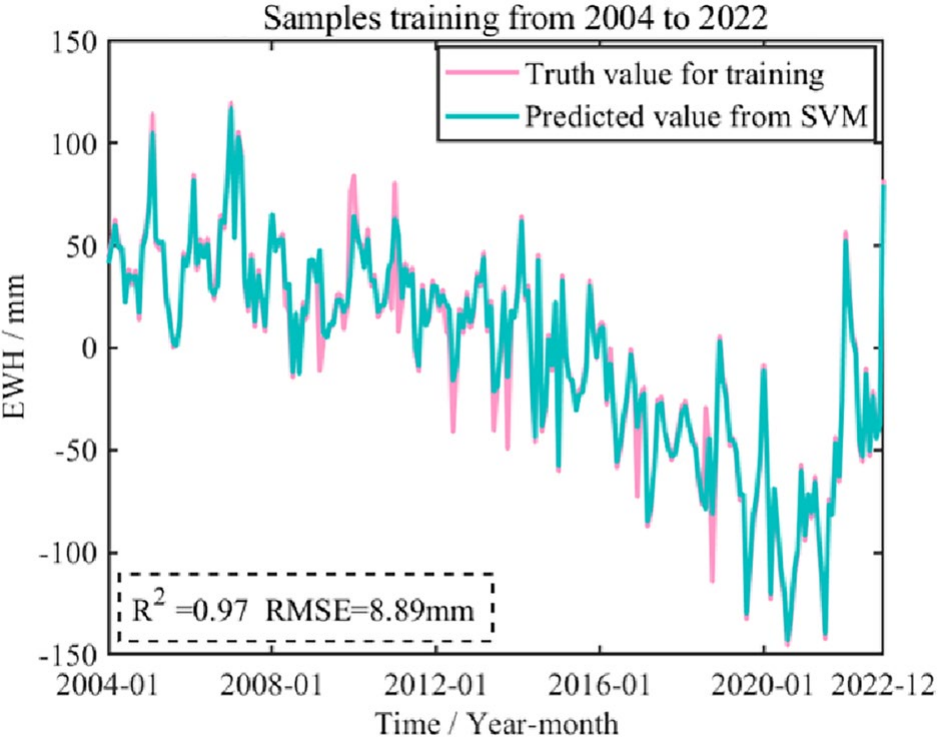
Predicted GWS change in Shandong Province from January 2023 to December 2023 from SVM.

Based on the above performance evaluation on short-term prediction from SVM, SVM method is adopted to train and model the time series of GWS changes in Shandong Province from January 2004 to December 2022. The number of training samples used in this paper is 228 months. The predicted signal from SVM and actual value for training from GRACE are shown as blue and red curves in Fig. 18.

It can be seen from Fig. 18 that the predicted signal from SVM modeling has a high degree of agreement with the original signal. Both *R*^2^ reaches 0.97, *RMSE* is 8.89 mm, indicating that SVM model based on training samples with 228 months can better reflect the characteristics of GWS changes in Shandong Province.

## Discussions

1. The limitation of each dataset
  There are limitations in the inversion of GWS changes in Shandong Province depending on GRACE data combined with hydrological models. On the one hand, it comes from the low spatial resolution with about 330 km of GRACE^33,34^, on the other hand, it is caused by the uncertainty of GLDAS and WGHM models. Due to the lack of measured data in some areas during the construction of the GPCP model, the model also has uncertainty in the local area. In addition, the GWS data from WGHM model also have limitations, which may lead to a significant difference between the results from GRACE and WGHM model during 2003 to 2006.
2. Error consideration
  There are errors in the spatio-temporal changes of GWS in Shandong Province derived from satellite gravity. On the one hand, it comes from the band noise existing in the time-variable gravity field model and filtering effects. In addition, leakage error is an essential source of error in GRACE inversion results^35^, which could be caused by leakage signal from GWS in Hebei Province monitored by GRACE.
3. Results interpretation
  The source of irrigation water is also crucial for GWS change. If the water source is local surface and ground water, and irrigation reduces the total amount of water in the study area. On the other hand, the water source comes from the outside of the study area such as the South to North water diversion project, the irrigation increases the amount of water in the study area. The impact of agricultural irrigation on GWS change in Shandong Province is relatively complex, the specific impact mechanism needs to be further studied (Supplementary information). It can be seen from the in-situ groundwater level and water volume change data in the water resources bulletin that there was a significant uplift in 2015, which may be owing to the South-to-North Water Diversion Project.
  The SVM method is used to train and model the sample data with only 228 months, however, the uncertainty of predicted results is not given. 204 and 180-month data can also be used for training and prediction. Because the pure mathematical method is adopted for predicting GWS in this paper, in the following research, the hydrological and meteorological data, such as precipitation, evapotranspiration, temperature, runoff and other data, can also be added to the external constraints, to provide the accuracy of prediction. The SVM network structure is used to establish the optimal prediction model, which can realize the short-term prediction of the results derived from GRACE. However, regarding medium and long-term prediction, the SVM algorithm has certain limitations. Based on the different number of training samples, the prediction results from SVM, LSTM, SSA and ARMA methods are compared and analyzed. It can be seen that the superiority of SVM model, but the prediction accuracy still needs to be improved. There will be some uncertainty in predicting GWS changes based on GRACE data.
4. Comparison with other studies
  Evapotranspiration is an essential part of the terrestrial water cycle. Climate warming increases potential evapotranspiration. Existing research results have proved that surface evapotranspiration caused by climate warming accelerates the consumption of groundwater resources in the United States^36^. Therefore, the impact of evapotranspiration on the change of GWS in Shandong Province should be considered, which will be improved in the subsequent study. In this paper, the research area is selected as Shandong Province in China. For areas with small spatial scales, it is difficult to effectively reveal the detailed characteristics of water storage changes by using GRACE data alone. Recently, many studies have used artificial intelligence algorithms such as machine learning and deep learning, combined with hydrometeorological data, to carry out downscaling processing to improve the spatial resolution of monitoring results from GRACE. The downscaling method for the results derived from GRACE will also be considered in future work.

## Conclusion

The GWS change derived from GRACE data in Shandong Province can be analyzed, compared with WGHM, GPCP, in-situ groundwater level, etc. The prediction of GWS change in Shandong Province under the deep learning framework is studied. The results show that SVM method have higher prediction accuracy than that from LSTM, SSA and ARMA model for short-term prediction. The specific conclusions are as follows:

1. The loss intensity of GWS in the west of Shandong Province decomposed by the ICA method is significantly greater than that in the coastal areas. The linear change rate of GWS from 2003 to 2006 is 9.84 ± 6.35 mm/a; there is an apparent loss in the GWS from GRACE. Its linear rate during 2007 to 2014 is − 5.80 ± 2.28 mm/a; the trend of GWS from GRACE loss weakened, linear rate during 2015 to 2022 is − 5.39 ± 3.65 mm/a, which may be owing to the effect of the South-to-North Water Diversion Project. However, after 2014, the loss trend of GWS continued to exist until 2019, from 2020 to 2021, GWS rebounded sharply, and the specific reasons need to further study.
2. The correlation coefficient between the overall GWS change derived from GRACE and WGHM model is 0.67. The annual trend of GWS is consistent with in-situ groundwater volume and level. The correlation coefficient between GWS and monthly precipitation reaches 0.23 after correcting time delay and smoothing average, which is also consistent with annual precipitation anomaly using CWT method. The variation of GWS derived from GRACE has a strongly correlates with the in-situ groundwater mining, and water consumption for agricultural irrigation. The correlation coefficients are 0.80 and 0.71, respectively. It shows that groundwater mining is the main factor affecting the change of GWS, followed by farmland irrigation water.
3. Based on the training samples of GWS change with different numbers of 228, 204 and 180 months, *R*^2^ between the predicted signal and training samples depending on SVM method is above 0.97. Compared with LSTM, ARMA, SSA methods, the accuracy index from SVM method is superior, indicating that SVM model has higher prediction accuracy for GWS change.

### Supplementary Information

Supplementary Information.

## Data Availability

All data generated or analysed during this study are included in this published article [and its supplementary information files].
