# Supplementary material for: The analysis on groundwater storage variations from GRACE/GRACE-FO in recent 20 years driven by influencing factors and prediction in Shandong Province, China
Source: Sci Rep. 2024 Mar 9;14:5819. doi: 10.1038/s41598-024-55588-3 (PMC10925065; doi:10.1038/s41598-024-55588-3)
Supplement: Supplementary file 1 — Supplementary Information. [file 41598_2024_55588_MOESM1_ESM.zip › data/Data declaration.docx]

**Data introduction**

The attachment provides all the data used in the article. The file named' allTimeseries2003-2022.txt' represents the time series of groundwater storage changes retrieved by satellite gravity and has been filled with discontinuities.

The file named 'GPCCTimeseries2003-2020.txt' represents the precipitation time series obtained from the GPCC precipitation model.

The file named 'GPCTimeseries2003-2020r.txt' represents the precipitation time series obtained by the GPCC precipitation model, but this sequence is processed by moving average and the abrupt signal is filtered out.

The file named 'recTimeseries2017-2018.txt' represents the time series of two generations of gravity satellite intermittent terrestrial water storage change products provided by the Qinghai-Tibet Plateau Scientific Data Center.

The file named 'soilTimeseries2017-2018.txt' represents the time series of soil water storage changes obtained by the GLDAS hydrological model.

The file named 'Timeseries2003-2022y.txt' represents the annual change value of the time series of groundwater storage change inverted by satellite gravity. This data is processed by monthly time series.

The file named 'WghmTimeseries2003-2019.txt' represents the time series of groundwater storage changes obtained by the WGHM model.

The document named 'Water Bulletin of Shandong Province.xlsx' represents the annual variation data of various types of water in Shandong Province published in the Shandong Provincial Water Resources Bulletin.
